# Application of Peplau's interpersonal relationship theory combined with case management in rehabilitation of patients after primary percutaneous coronary intervention

**DOI:** 10.3389/fcvm.2024.1420974

**Published:** 2024-10-24

**Authors:** Qin Lu, Zhenliang Chu, Yeping Zheng, Juanqin Shen, Jingjing Lu, Jianjiang Xu, Songchao Wang

**Affiliations:** ^1^ Department of Cardiology, The Second Affiliated Hospital of Jiaxing University, Jiaxing, Zhejiang, China; ^2^ Health Management Center, The Second Affiliated Hospital of Jiaxing University, Jiaxing, Zhejiang, China; ^3^ Department of Nursing Administration, The Second Affiliated Hospital of Jiaxing University, Jiaxing, Zhejiang, China; ^4^ Department of Hospital Quality Management, The Second Affiliated Hospital of Jiaxing University, Jiaxing, Zhejiang, China

**Keywords:** Peplau’s interpersonal relationship theory, case management [MeSH], percutaneous coronary intervention, exercise-based cardiac rehabilitation, coronary heart disease (CAD)

## Abstract

**Objective:**

To explore the effect of Peplau's interpersonal relationship theory (PIRT) combined with case management (CM) on exercise-based cardiac rehabilitation (EBCR), self-efficacy of rehabilitation and risk factors in patients after primary percutaneous coronary intervention (PCI).

**Methods:**

The convenience sampling method was used to select patients who were admitted to the Department of Cardiology in our hospital from January to October 2022 and received PCI for the first time. Patients were divided into a control group and an intervention group. The control group was given routine treatment and health guidance, including radial artery puncture site care, monitoring of vital signs, informing about medication and dietary precautions, etc. and the intervention group was given PIRT combined with CM. The study was conducted for 3 months. The effect of intervention in the two groups was evaluated by the coronary heart disease risk factor index, EBCR knowledge-attitude-behavior questionnaire and EBCR self-efficacy scale.

**Results:**

The rate of risk factors control including blood pressure (*p* < 0.001), low density lipoprotein cholesterol (*p* = 0.012), smoking cessation (*p* = 0.031) and exercises (*p* = 0.021), the scores of EBCR knowledge (*p* < 0.001), attitude (*p* = 0.001) and behavior (*p* < 0.001), and the score of EBCR self-efficacy scale (*p* < 0.001) in the intervention group were better than those in the control group at 3 months after intervention.

**Conclusion:**

Peplau's interpersonal relationship theory combined with appropriate case management can effectively control cardiovascular disease in patients after primary PCI.

## 1 Introduction

Cardiac rehabilitation (CR), especially exercise-based cardiac rehabilitation (EBCR), following percutaneous coronary intervention (PCI) has attracted the attention of scholars from China and other countries (1–5). As an important part of modern CR, EBCR can restore functional status, increase cardiopulmonary exercise tolerance in patients with coronary heart disease (6) improve exercise capacity, fundamentally remove risk factors for coronary heart disease, maintain the patency of coronary artery lumen, improve quality of life, and reduce mortality (7). Therefore, it is increasingly important to implement CR in the treatment of patients with primary PCI. Peplau's theory centers on interpersonal relationships, promoting positive interactions between medical staff and patients, and it proposes more optimized personalized care methods through communication, aiming to achieve emotional satisfaction and skill development for both parties in the interpersonal communication process (8). Case management (CM) is a comprehensive and continuous care service that integrates assessment, planning, implementation of care, coordination, and monitoring (9). It seeks to balance cost-effectiveness and quality of care by reducing costs and shortening hospital stays. This study explores the application of Peplau's interpersonal relationship theory (PIRT) combined with appropriate CM in patients after primary PCI and its influence on risk factor, and knowledge-attitude-behavior (KAB) questionnaire and self-efficacy scale of EBCR.

## 2 Research objects and methods

### 2.1 Subjects

We estimated a sample size of 115 patients by sample size calculation formula (10), and these patients who underwent PCI for the first time in the Department of Cardiology of in our hospital from January to October 2022 were selected for inclusion in the study. The patients were divided into a control group and an intervention group according to the random number table method by research assistant. The inclusion criteria were as follows: (1) Patients who met the diagnostic criteria for coronary heart disease stipulated in the 2016 edition of the guidelines (11)and were recommended for CR after undergoing PCI for the first time; (2) Complete cardiopulmonary exercise test or 6-minute walk test assessment before discharge; (3) Can understand the content of the scale, communication barrier-free; (4) Skilled in the use of smart devices; (5) Provide informed consent and voluntary participate in this study; (6) Patients with an urban residence. The exclusion criteria were as follows: patients with severe physical diseases and complications, mental and psychological disorders, motor dysfunction, and inability to regularly complete the scale survey. Patients were excluded from the study analysis for the following reasons: (1) Patients, due to personal reasons, no longer wish to continue and requested to withdraw from the study; (2) Lost contact with patients for various reasons; (3) Patients refused the questionnaire survey; (4) The recovery questionnaire was unqualified, and the obvious regular answers or missing items exceeded 20% of the questionnaire. This study was approved by the ethics committees of the Second Affiliated Hospital, Jiaxing University (Ethics number: JXEY-2022SW083), which accorded with the ethical standards formulated in the Helsinki Declaration. All the patients offering clinical materials were signed the informed consents.

### 2.2 Methods

#### 2.2.1 Sample size calculation

**Figure 1:**
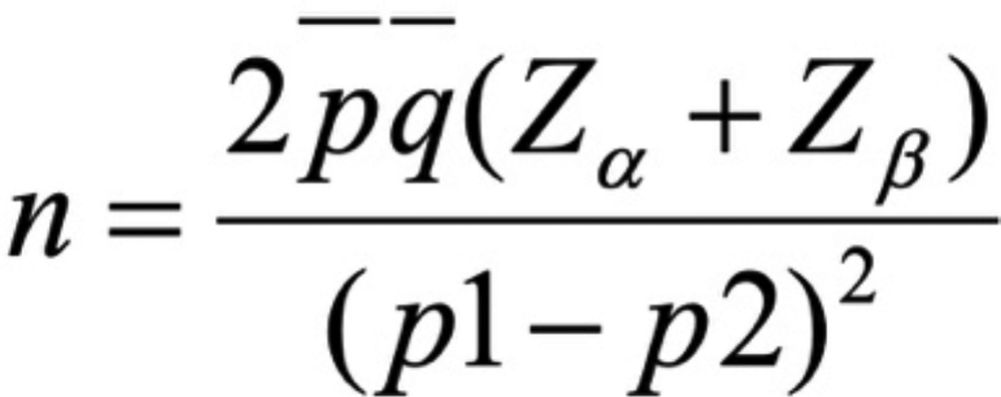
Sample size calculation formula.

We used the sample size calculation formula (10) to determine the sample size (Figure 1). Based on preliminary pilot observations of 10 cases in each group, the effectiveness rate of LDL-C treatment in the control group was 53.5%, while in the intervention group it was 82%. Typically, with *α* set at 0.05 and a two-sided Z value (Z_0.05_) of 1.96, and with β being one-sided and the power (1-β) at 0.9, the Z_β_ value is 1.28. Let p1 and p2 represent the estimated proportions for the two groups, respectively. $\bar{p}$ is the average of p1 and p2, and $\bar{q}$ is the average of 1-p1 and 1-p2. By substituting these values into the sample size formula and considering a 10% sample attrition rate, the total sample size was determined to be 115.

#### 2.2.2 Control group

The patients in the control group received routine treatment and health guidance after coronary stenting, including: (1) radial artery puncture site care and monitoring of vital signs; (2) medication management, informing about medication as prescribed, adhering to the prescribed dosage and schedule without altering the dose independently, and informing about the side effects and precautions of the medications; (3) dietary adjustments, focusing on light, easily digestible, and nutritious foods, maintaining regular eating habits, and avoiding overeating; (4) activity guidance, gradually performing EBCR based on individual conditions, avoiding high-intensity activities; (5) mental health, actively adjusting one's mindset, and maintaining an optimistic and positive attitude; (6) follow-up visits, regularly following up and revisiting as advised by the doctor; (7) before discharge, informing patients about potential unexpected situations that may occur during exercise and the appropriate ways to handle them; (8) recording follow-up status and addressing their questions via phone and WeChat app at 2 weeks, 1 month, and 3 months after PCI.

#### 2.2.3 Intervention group

On the basis of the control group's treatment measures, the intervention group patients received treatment according to PIRT combined with CM (8, 9). This included establishing health records for post-PCI patients, which encompassed information such as age, gender, ethnicity, medical history, family history, insurance status, household income, general information, disease condition, and distributing “Self-Management Manual of PCI” designed by department of cardiology of our hospital. Specific interventions were as follows:

(1) Team formation

The team included one CR specialist, one case manager, and three specialized nurses. Selection criteria for the case manager: Bachelor's degree or higher; At least 5 years of experience in the cardiovascular field; Completion of systematic training and assessment in CM and PIRT with proficiency in these methods. Job responsibilities: The CR specialist was responsible for patient enrollment; the case manager was responsible for assessing the health needs of patients after PCI and formulating health plans, coordinating the work distribution of members in the group, and guiding patients to communicate with WeChat; the specialist nurses were responsible for implementing the health plan.

(2) Evaluation: orientation phase of PIRT

A close nurse-patient relationship is established with good communication. After PCI, while focusing on the patient's condition, it was important to understand their basic information and think from the patient's perspective. This involved using appropriate communication methods to build trust, allowing the patient to accept and cooperate with the nursing interventions.

(3) Planning: Confirmation Phase of PIRT

In this phase, the relationships among the doctor, nurse, and patient are characterized by interdependence, passive dependence, and independence, respectively. Interdependence is best demonstrated in the nurse‒patient relationship. Patients often believe that PCI will greatly affect their normal life in the future, and they display negative emotions such as anxiety and depression. Patients should be made aware of the importance of healthy lifestyles for disease outcomes. Professional assessment: Professional assessment tools, such as the patients’ KAB and self-efficacy scale of EBCR, require patients and their families to complete the scale together for evaluation by case managers. Based on the results of basic and professional assessments, a CM plan in line with the actual situation of patients is formulated. Patients and their families are required to participate in the CM plan, popularize the relevant knowledge after PCI by WeChat, and ensure that patients participate in the plan formulation.

(4) Implementation: Exploitation Phase of PIRT

In this phase, the focus is on providing patients with comprehensive preoperative education and preparation by the nursing staff, as well as teaching them methods to prevent postoperative complications. At the same time, further details about the patient's exercise habits, family situation, and other relevant factors should be gathered. Habits that are beneficial to the patient's health behavior should be guided and encouraged, while those that negatively impact health behavior should be explained and corrected. This helps to stimulate the patient's interest in self-management. If the patient shows signs of non-cooperation, the nursing staff should use understanding language to actively persuade them. Exercise management: Based on the medical examination data, such as exercise tests and physical fitness tests, as well as the health, physical strength and cardiovascular function of patients, CR specialists recommended moderate aerobic activities, such as swimming, cycling, Tai Chi, square dance, Baduanjin, etc. Exercise intensity is prescribed in a personalized manner based on target heart rate and perceived exertion values. For example, the target heart rate is controlled as follows: target heart rate = peak heart rate × (60%–80%), and the Borg Rating of Perceived Exertion (RPE) is 12∼14 (12). The exercise duration includes a warm-up of 5–10 min, training for 30–40 min, and a cool-down of 5–10 min, with a frequency of 3–5 times per week (12). Additionally, precautions for the exercise process are provided.

(5) Solution Phase of PIRT

The purpose of this phase is to evaluate whether the nursing care has met the required standards. For those who meet the standards, encouragement and praise are given. For those who do not, the reasons need to be promptly understood, and the CM plan should be adjusted accordingly. At 3 months post-discharge, changes in risk factor indicators are collected, and the patient's understanding of CR content and self-efficacy in EBCR are assessed. The follow-up period is determined, and the case is closed with confirmation from the case manager.

### 2.3 Evaluation indicators

(1) Self-efficacy in exercise

Developed by Hickey (13), introduced by Chinese scholar Sun Yuxiao (14), and translated into Chinese, the “Self-Efficacy Scale for EBCR” is a single-dimensional questionnaire comprised of 16 items which has good internal consistency and stability. Using the Likert 5-level scoring method, “very weak confidence” to “strong confidence” was indicated by a score of 1–5, respectively. The total score range was 16–80 points. The higher the score, the higher the self-efficacy of EBCR. The Cronbach's α coefficient of the scale was 0.90, and the retest correlation coefficient was 0.87.

(2) Knowledge- Attitude-Behavior questionnaire of EBCR

The “ KAB Questionnaire of EBCR in Coronary Heart Disease Patients” developed by Zhao Mengli (15) consists of three dimensions: knowledge, attitude, and behavior, with 12, 6, and 5 items respectively, for a total of 23 items. Each item was answered with “yes” or “no” based on the respondent's awareness. The awareness rate reflects the perceived status of knowledge, attitude, and behavior regarding EBCR among the study subjects. The Cronbach's *α* coefficient of the questionnaire was 0.833, and the Cronbach's α coefficients of the knowledge, attitude and behavior dimensions were 0.733, 0.707 and 0.752, respectively.

(3) Control of Risk Factors

The coronary heart disease risk factors included blood pressure (BP), low density lipoprotein cholesterol (LDL-C), fasting plasma glucose (FPG), smoking, exercise and body mass index (BMI). The following evaluation criteria were implemented according to the American College of Cardiology/American Heart Association (ACC/AHA) and Chinese guidelines (16, 17): (1) BP < 140/90 mmHg or <130/80 mmHg for patients with diabetes; (2) LDL-C <1.8 mmol/L; (3) FPG < 6.1 mmol/L; (4) completely quit smoking and avoid passive smoking; (5) the time of moderate intensity aerobic exercise ≥150 min per week excluding daily work and life activities; (6) BMI 18.5–24.9 kg/m^2^. If the above standards were met, the control of risk factors was considered to be satisfactory; otherwise, it is considered unsatisfactory.

### 2.4 Data collection and collation

Unified standards were adopted for the evaluation indicators to be measured to reduce the bias of the measurement results. The collected questionnaires were checked to ensure the integrity and effectiveness of the questionnaire. Before data entry, each questionnaire was checked twice. If questionnaires were not completed, this was discussed with patients on the spot, and the questionnaire data were completed. Patients who did not understand the contents of the questionnaire were excluded from analysis.

### 2.5 Statistical methods

SPSS 22.0 statistical software was used for data analysis. Data were expressed as mean ± standard deviation for normally distributed variables. Categorical data were expressed as numbers (%). When necessary, variables were transformed for further analyses. Group differences were evaluated using Student *t*-tests or Mann-Whitney *U*-tests for continuous variables and chi-square or Fisher exact tests for categorical variables. A value of *p* < 0.05 was considered statistically significant.

## 3 Results

### 3.1 General information

**Figure 2:**
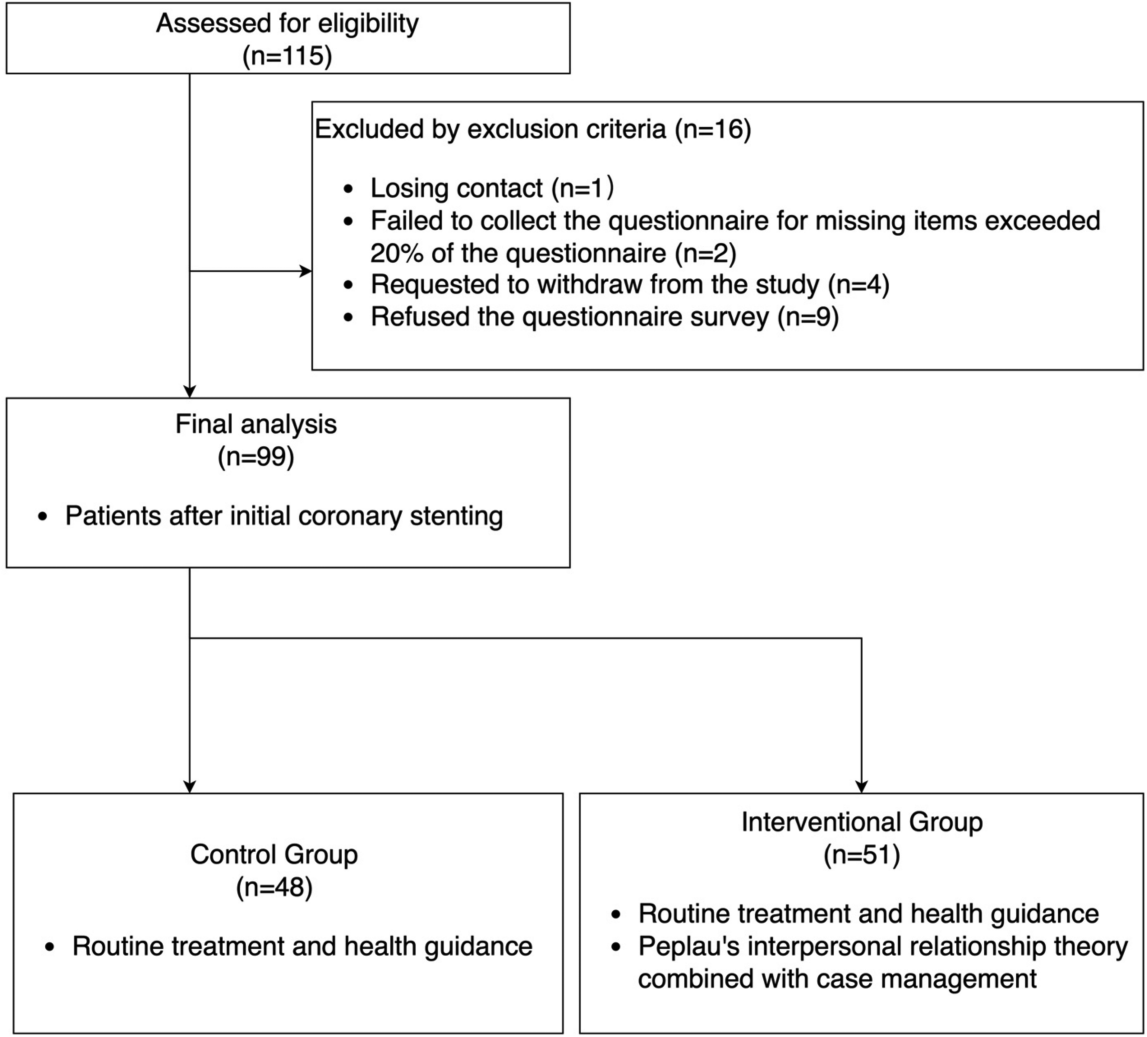
The CONSORT flow chart.

During the study period, 115 patients meeting inclusion criteria were included in the study (Figure 2). 16 patients met exclusion criteria. Finally, 48 patients and 51 patients were included in control group and intervention group respectively and completed this study without any adverse events. There was no significant difference in the general information between the two groups (*p* > 0.05) (See Table 1).

**Table 1:** Comparison of two groups of general information.

Groups	*n*	Age (year)	Gender		Standard of culture		Hypertension	Diabetes	Hyperlipidemia
			Male	Female	Primary school and below	Middle school and above			
Control group	48	59.73 ± 13.42	32	16	7	41	24	12	10
Interventional group	51	56.69 ± 12.92	35	17	8	43	25	13	9
*t*/*χ*^2^		1.150	0.005		0.023		0.009	0.003	0.162
*p*		0.253	0.947		0.878		0.922	0.955	0.687

### 3.2 Exercise self-efficacy

There were statistically significant differences in the scores of exercise self-efficacy between the two groups at 3 months after discharge (*p* < 0.05), as shown in Table 2.

**Table 2:** Comparison of exercise self-efficacy between the two groups before and after intervention.

Items	Before intervention				After intervention			
	Control group (*n* = 48)	Interventional group (*n* = 51)	*t*	*p* for inter-group	Control group (*n* = 48)	Interventional group (*n* = 51)	*t*	*p* for inter-group
1	3.29 ± 1.11	3.49 ± 1.10	0.893	0.374	3.69 ± 1.01^a^	4.08 ± 0.74^b^	2.196	0.030^c^
2	3.33 ± 1.24	3.57 ± 1.17	0.970	0.335	3.81 ± 0.94^a^	4.25 ± 0.69^b^	2.687	0.008^d^
3	3.29 ± 1.05	3.53 ± 1.14	1.078	0.284	3.73 ± 0.74^a^	4.04 ± 0.72^b^	2.118	0.037^c^
4	3.69 ± 1.13	3.63 ± 1.08	0.270	0.787	3.94 ± 0.86^a^	4.25 ± 0.59^b^	2.145	0.034^c^
5	3.46 ± 1.11	3.35 ± 1.02	0.493	0.623	3.67 ± 0.88^a^	4.06 ± 0.81^b^	2.304	0.023^c^
6	3.58 ± 1.09	3.37 ± 1.00	1.344	0.318	3.79 ± 0.82^a^	4.20 ± 0.63^b^	2.748	0.007^d^
7	3.69 ± 1.27	3.76 ± 1.18	0.313	0.755	3.98 ± 0.91^a^	4.39 ± 0.57^b^	2.724	0.008^d^
8	3.52 ± 1.15	3.59 ± 1.13	0.294	0.770	3.79 ± 0.82^a^	4.18 ± 0.62^b^	2.630	0.001^d^
9	3.65 ± 1.14	3.57 ± 1.12	0.340	0.734	3.88 ± 0.84^a^	4.20 ± 0.57^b^	2.240	0.003^d^
10	3.56 ± 1.15	3.57 ± 1.14	0.027	0.979	3.79 ± 0.87^a^	4.20 ± 0.60^b^	2.696	0.008^d^
11	3.5 ± 1.11	3.41 ± 1.00	0.415	0.679	3.75 ± 0.81^a^	4.10 ± 0.5^b^	2.584	0.011^c^
12	3.48 ± 1.05	3.55 ± 1.12	0.320	0.750	3.71 ± 0.77^a^	4.20 ± 0.53^b^	3.688	<0.001^d^
13	3.50 ± 1.11	3.25 ± 1.04	1.136	0.259	3.75 ± 0.81^a^	4.06 ± 0.51^b^	2.285	0.025^c^
14	3.65 ± 1.14	3.51 ± 1.05	0.619	0.537	3.85 ± 0.87^a^	4.18 ± 0.48^b^	2.293	0.024^c^
15	3.42 ± 1.18	3.55 ± 1.22	0.547	0.585	3.73 ± 0.84^a^	4.29 ± 0.46^b^	4.168	<0.001^d^
16	3.44 ± 1.20	3.53 ± 1.19	0.382	0.703	3.73 ± 0.89^a^	4.29 ± 0.50^b^	3.910	<0.001^d^
总分	56.04 ± 4.50	56.24 ± 3.87	0.230	0.819	60.58 ± 3.60^a^	66.96 ± 2.06^b^	10.910	<0.001^d^

^a^ Intra-group comparison before and after intervention in control group, with *p* < 0.01.

^b^ Intra-group comparison before and after intervention in intervention group, with *p* < 0.01.

^c^ Inter-group comparison after intervention in intervention group, with *p* < 0.05.

^d^ Inter-group comparison after intervention in intervention group, with *p* < 0.01.

### 3.3 Knowledge, attitude and behavior score

There was no significant difference in the scores of KAB between the two groups before intervention (*p* > 0.05); after 3 months of discharge, the scores of all dimensions of KAB in the intervention group were higher than those in the control group, and the difference was statistically significant (*p* < 0.01) (see Table 3).

**Table 3:** Comparison of knowledge, attitude and behavior between the two groups before and after intervention.

Dimension (%)	Before intervention				After intervention			
	Control group (*n* = 48)	Interventional group (*n* = 51)	*χ* ^2^	*p* for inter-group	Control group (*n* = 48)	Interventional group (*n* = 51)	*χ* ^2^	*p* for inter-group
Knowledge (12 items)	52.26 (301/576)	48.20 (295/612)	1.951	0.162	76.56 (441/576)^a^	86.44 (529/612)^b^	19.314	<0.001
Attitude (6 items)	53.47 (154/288)	49.35 (151/306)	1.011	0.315	77.08 (222/288)^a^	87.25 (267/306)^b^	10.548	0.001
Behavior (5 items)	46.25 (111/240)	48.24 (123/255)	0.196	0.658	67.92 (163/240)^a^	85.10 (217/255)^b^	20.464	<0.001

^a^ Intra-group comparison before and after intervention in control group, with *p* < 0.001.

^b^ Intra-group comparison before and after intervention in intervention group, with *p* < 0.001.

### 3.4 Risk factors control

The rate of risk factors control including blood pressure (BP), low density lipoprotein cholesterol (LDL-C), smoking and lack of exercises in the intervention group were higher than those in the control group at 3 months after intervention (*p* < 0.05), as shown in Table 4.

**Table 4:** Comparison of risk factors control between the two groups before and after intervention (*n*,%).

Groups	*n*	BP<140/90 mmHg or <130/80 mmHg for patients with diabetes		LDL-C <1.8 mmol/L		FPG<6.1 mmol/L		Smoking cessation		Exercises		BMI 18.5–24.9 kg/m^2^	
		Before	After	Before	After	Before	After	Before	After	Before	After	Before	After
Control group	48	23 (47.92)	27 (56.25)	14 (29.17)	23 (47.92)	30 (62.5)	39 (81.25)^a^	23 (47.92)	28 (58.33)	10 (20.83)	19 (39.58)^a^	18 (37.5)	27 (56.25)
Interventional group	51	31 (60.78)	43 (84.31)^b^	16 (31.37)	37 (72.55)^b^	32 (62.75)	46 (90.2)^b^	26 (50.98)	40 (78.43)^b^	10 (19.61)	32 (62.75)^b^	20 (39.22)	36 (70.59)^b^
*X* ^2^		1.651	12.406	0.057	6.284	0.001	1.630	0.093	4.644	0.023	5.311	0.031	2.197
*p* for inter-group		0.200	<0.001	0.811	0.012	0.980	0.202	0.765	0.031	0.879	0.021	0.861	0.138

BP, blood pressure; LDL-C, low density lipoprotein cholesterol; FPG, fasting blood glucose; BMI, body mass index.

^a^ Intra-group comparison before and after intervention in control group, with *p* < 0.05.

^b^ Intra-group comparison before and after intervention in intervention group, with *p* < 0.01.

## 4 Discussion

In this study, the combination of PIRT and CM effectively improved the KAB and self-efficacy of EBCR and risk factors control in patients after PCI. The postoperative rehabilitation of PCI patients focused on control of the risk factors for coronary heart disease. A scientific exercise prescription is a key element in ensuring the success of EBCR. It helps patients exercise effectively and achieve their rehabilitation goals.

The KAB theory posits that health knowledge and information can help individuals establish positive and correct attitudes and beliefs, which in turn drive behavioral change, and the motivation for such change stems from these attitudes and beliefs (15). The more positive an individual's attitude, the better their subjective behavioral norms, the stronger their perceived behavioral control, and the stronger their behavioral intentions. Consequently, the likelihood of adopting the corresponding behaviors increases. Sitot et al. (18) thought that according to the KAP theory, individuals can only develop healthy behaviors by acquiring relevant health knowledge and establishing positive beliefs and attitudes. The study by Kirchberger (19) indicated that the use of cardiac rehabilitation CM for elderly patients with acute myocardial infarction can significantly improve their functional status. These findings are consistent with the results of this study, which indicate that using PIRT combined with CM is superior to conventional nursing measures in the control group in terms of improving patients’ knowledge, attitudes, and behavior of EBCR, as well as their self-efficacy scores.

In this study, the application of PIRT combined with CM strengthened the therapeutic interaction between healthcare providers and patients, establishing a good nurse-patient relationship. The intervention was carried out in four stages, fully considering the physiological and psychological changes of the patients. During the orientation and confirmation phases, establishing a good relationship between healthcare providers and patients had a positive impact on subsequent interventions. In the exploitation phase, healthcare providers stimulated patients’ interest in self-management after PCI, improving their knowledge, attitudes, and behaviors of EBCR, as well as their self-efficacy. In the resolution phase, patients received further guidance to help them move beyond dependency, face their disease and life with a better attitude, and gradually improve their exercise self-efficacy.

The case manager's health education for patients was continuous, repetitive, and reinforced the patients’ health concepts. Both the healthcare providers and patients need to maintain mutual respect, understanding, and participation in nursing activities (20). Family members and friends were encouraged to join in the exercises with the patients to increase their exercise enthusiasm. Peplau's Interpersonal Relations Theory focuses on the relationship between nurses and patients, viewing nursing as an interpersonal relationship within a specific framework (21). Interventions based on PIRT combined with CM can effectively improve exercise self-efficacy in patients after their first PCI. This model enables comprehensive assessment of the patient, allows healthcare providers and patients to jointly develop an exercise intervention plan, and provides targeted care, thereby helping to enhance the patient's exercise self-efficacy.

The effectiveness of PIRT combined with CM in intervening on risk factors for patients after their first PCI was examined. Comprehensive assessment of multiple risk factors is a routine strategy for post-PCI patients. Hypertension, hyperlipidemia, hyperglycemia, smoking, lack of exercise, and obesity are major risk factors for the development of coronary heart disease and affect the prognosis of patients with coronary heart disease. Post-PCI cardiac rehabilitation is a comprehensive program based on exercise, combined with education and behavior modification, to help patients develop good lifestyle habits and improve self-management skills, which positively impact the control of coronary heart disease risk factors. This study found that the control rates of risk factors (blood pressure, LDL-C, smoking, exercise) in the intervention group were higher than those in the control group, suggesting that the intervention strategy using PIRT combined with CM can effectively control cardiovascular disease risk factors. This is consistent with the findings of Xiao lina (22), who applied a CM model to intervene with post-PCI patients for 3 months, concluding that CM can improve patients’ self-management abilities and health behaviors. Ishani (23) focused on the impact of nurse-led CM on cardiovascular risk factors in diabetic patients. The case manager and patients jointly developed detailed management goals and personalized action plans, and the results showed that the CM method could effectively improve cardiovascular risk factors in patients, consistent with the results of this study. The possible reasons are: (1) The CM team members help patients master relevant knowledge, enabling them to comprehensively and correctly understand cardiovascular diseases, which helps in controlling and preventing cardiovascular disease risk factors; (2) The healthcare providers and patients jointly formulate an exercise self-management plan, using exercise tracking to understand patients’ exercise status. The CM team members implement targeted exercise intervention measures, helping patients develop good exercise habits, control disease progression, and effectively reduce cardiovascular disease risk factors. Marchese (24) used the concept of mutual participation from PIRT to establish a 5-year therapeutic relationship and provide CM for a bladder cancer patient. Despite the patient's persistent concerns about cancer recurrence, sexual dysfunction, and changes in body image, the patient was able to achieve the best possible health status.

Through the nursing intervention based on PIRT combined with CM, this study encouraged patients to express their inner feelings, which helped nurses identify patients’ inappropriate cognition and then help them correct and reconstruct correct cognition. According to the actual situation of the patients, an easy-to-accept intervention method was adopted to help the patients enhance their confidence in overcoming the disease so that the patients could actively face difficulties and accept the changes in their body and life, thus effectively improving the quality of life of the patients. However, the patients’ education level, economic level and personality characteristics were different. The case manager fully considers the patient's physiological, psychological and social conditions when formulating measures to improve patient compliance and formulates practical interventions based on the patient's living environment to improve patient compliance. Long-term follow-up can enable case managers to understand the implementation of the plan, identify problems, and solve problems.

In conclusion, the application of PIRT combined with CM in patients after their first PCI can improve the control of cardiovascular disease risk factors, the willingness to participate in cardiac rehabilitation, and adherence to exercise, thereby enhancing exercise self-efficacy.

This study has some limitations. The first is small sample size. The sample for this study was only drawn from a single center, resulting in a small sample size with limited representativeness. Future studies with multicenter, large samples are necessary to further validate this conclusion. The second is short intervention period. This study did not conduct long-term follow-up, so the long-term effects of applying PIRT combined with CM remain unclear. Further tracking and follow-up are needed in future studies to confirm this conclusion.

## Data Availability

The original contributions presented in the study are included in the article/Supplementary Material, further inquiries can be directed to the corresponding author.
